# Dietary fermented products using *koji* mold and sweet potato-*shochu* distillery by-product promotes hepatic and serum cholesterol levels and modulates gut microbiota in mice fed a high-cholesterol diet

**DOI:** 10.7717/peerj.7671

**Published:** 2019-09-12

**Authors:** Toshiki Kosakai, Hirotaka Kato, Cho Sho, Kuniaki Kawano, Ken-ichi Iwai, Yoshikazu Takase, Kenjiro Ogawa, Kazuo Nishiyama, Masao Yamasaki

**Affiliations:** 1 Interdisciplinary Graduate School of Agriculture and Engineering, University of Miyazaki, Miyazaki, Miyazaki, Japan; 2 Kirishima Shuzo Co., Ltd., Miyakonojo, Miyazaki, Japan; 3 Graduate School of Agriculture, University of Miyazaki, Miyazaki, Miyazaki, Japan; 4 Organization for Promotion of Tenure Track, University of Miyazaki, Miyazaki, Miyazaki, Japan

**Keywords:** Sweet potato-*shochu*, Distillery by-product, Dietary fiber, Short chain fatty acids, Gut microbiota, Koji, Cholesterol

## Abstract

It has been reported that fermented products (FPs) prepared from sweet potato-*shochu* distillery by-product suppressed weight gain and decreased serum cholesterol levels in mice under normal dietary conditions. Furthermore, from the information gained from the above data regarding health benefits of the FPs, the aim of this study was evaluating the effects of dietary FPs on lipid accumulation and gut microbiota in mice with or without cholesterol-load in the diet. C57BL/6N mice were fed normal (CO) diet, CO with 10% FPs (CO + FPs) diet, cholesterol loaded (HC) diet, or HC with 10% FPs (HC + FPs) diet for 8 weeks. The mice were then euthanized, and blood samples, tissue samples, and feces were collected. The adipose tissue weight and liver triglyceride levels in the HC + FPs diet groups were significantly reduced compared to that in the HC diet groups. However, FPs significantly increased the serum non-high-density lipoprotein cholesterol (HDL-C) levels, the ratio of non-HDL-C to HDL-C and hepatic total cholesterol levels in mice fed cholesterol-loaded diet compared with that of the HC diet group. Since dietary FPs significantly decreased the protein expression levels of cholesterol 7 alpha-hydroxylase 1 in the HC + FPs diet groups, the cholesterol accumulation in FPs group may be explained by insufficient catabolism from cholesterol to bile acid. In addition, the dietary FPs tended to increase *Clostridium* cluster IV and XIVa, which are butyrate-producing bacteria. Related to the result, *n*-butyrate was significantly increased in the CO + FPs and the HC + FPs diet groups compared to their respective control groups. These findings suggested that dietary FPs modulated the lipid pool and gut microbiota.

## Introduction

Obesity is caused by an imbalance between energy intake and energy consumption (Spiegelman & Flier, 2001), and it is a risk factor for the disruption of lipid metabolism (Furukawa et al., 2017). In Japan, the clinical criteria of metabolic syndrome include abnormal values for more than two of the following parameters: weight, triglycerides (TG), high-density lipoprotein (HDL) cholesterol in serum, and blood pressure (Alberti et al., 2009). Metabolic syndrome related to the disruption of lipid metabolism increases the risk of cardiovascular disease and type 2 diabetes (Grundy, 2016; DeBoer et al., 2016). Metabolic syndrome is a growing social problem, and the efficacy of various functional foods such as green tea (Suzuki et al., 2013), coffee (Watanabe et al., 2017), and soybean (Hashidume et al., 2016) on metabolic syndrome has been evaluated. It is expected that food materials having preventive effects on the disruption of lipid metabolism will be developed in future.

*Shochu* is a traditional Japanese liquor and is one of the distilled spirits made from sweet potato, barley, rice, buckwheat, and brown sugar (Sameshima, 2004). The sweet potato-*shochu* distillery by-product (SSDB) discharged from *shochu* industry is utilized as materials for methane fermentation or animal feed (Kamizono et al., 2010). It is reported that the SSDB contains various nutritional components such as proteins, vitamins and minerals, and functional components such as S-adenosylmethionine and polyamines (Mukai et al., 2017). It has been reported that SSDB has an anti-cancer effect (Sasaki et al., 2005). Among them, by-products of *awamori* (one of the traditional Japanese spirits) lowered serum total cholesterol (TC) levels (Ishibashi, 2007; Nohara et al., 2010). Moreover, it is used for the preparation of bread (Sho et al., 2008) and vinegar manufacture (Kawano et al., 2008), and also for the production of chitosan (Yokoi et al., 1998) and nisin (Furuta et al., 2008).

*Koji* is cultured by solid fermentation using *koji* molds on rice and barley. Because *koji* mold produces many types of enzymes and secretes them as it grows, it is known that the *koji* mold is traditionally used for solid fermentation to produce fermented foods like *shochu*, *sake* (Japanese rice wine), *miso* (fermented soybean paste), soy source, and vinegar. Among them, because of the long experience of eating in Japan, *koji* mold is one of the safest microorganisms. It was indicated that dietary rice *koji*-fermented product produced by using *koji* mold reduced weight gain and adipose tissue in mice (Yoshizaki et al., 2014). Yoshimoto et al. (2004) reported that SSDB treated with *Bacillus subtilis* and cellulase is rich in caffeic acid, one of the many polyphenols that has beneficial physiological functions. In addition, glucosylceramide contained in the *koji* mold is capable of acting as a prebiotic (Hamajima et al., 2016), and barley-derived β-glucan improves metabolism in mice fed high-fat diet through short chain fatty acids (SCFAs) produced by intestinal bacteria (Miyamoto et al., 2018). It is also observed that *Aspergillus* species also contain polysaccharides, such as β-glucan (Ishibashi et al., 2004). Furthermore, it has been reported that the intestinal microbiota is altered by a high cholesterol diet (Caesar et al., 2016; Bo et al., 2017) and that SCFA production is increased by dietary fiber intake (Lattimer & Haub, 2010; Slavin, 2013). As described above, FPs are rich in polysaccharides, and we hypothesized that it would benefit cholesterol metabolism through changes in intestinal microbiota and SCFA production.

The aim of this study was evaluating the effects of dietary FPs on lipid accumulation and gut microbiota in mice with or without cholesterol-load in the diet. To the best of our knowledge, our study is the first to report the effects of FPs on cholesterol loading.

## Materials and Methods

### SSDB production

We recovered SSDB derived from *shochu* process after atmospheric distillation in Kirishima Shuzo Co., Ltd. (Miyazaki, Japan). SSDB was filtered through a filter press 40D-8 (Yabuta industries Co., Ltd., Hyogo, Japan). The filtrate was preserved at −20 °C and thawed prior to use. Kawachi NK, black *koji* of NK type, was purchased from Kawachi Gen-ichiro store Co., Ltd. (Kagoshima, Japan).

### Preparation of FPs made from SSDB using *koji* mold

To prepare the FPs, we followed a previously reported method (Kosakai et al., 2019). Kawachi NK (2 g) was suspended in 20 mL of sterile distilled water by vortexing and ultrasonication. SSDB (4 L) was poured into a 10 L jar fermenter (MDL-10 L; B. E. Marubishi Co., Ltd., Tokyo, Japan) and 3 × 10^5^ conidiospores per mL of medium were inoculated. The culture was incubated at 30 °C at 250 rpm without aeration for 48 h, followed by a rotation speed of 200 rpm at an aeration rate of 0.5 vvm for 48 h. FPs were recovered by suction filtration through a filter paper (No. 2; Toyo Roshi Kaisha, Ltd., Tokyo, Japan), and were freeze-dried and powdered. The experiment procedure from liquid-fermentation to powdering was repeated 18 times, mixed and used for performing animal experiments.

### Nutrient value of FPs

The nutrient contents, such as moisture, protein, fat, ash, carbohydrates, dietary fiber, and energy of FPs were analyzed by Japan Food Research Laboratories (Tokyo, Japan) in accordance with Cabinet Office Ordinance No. 10 of 2015 in Japan. Moisture and protein were measured by heating-drying method and combustion method, respectively. Fat and ash were measured gravimetrically after the extraction and the incineration, respectively. Carbohydrate was calculated by subtracting the amount of moisture, protein, fat, and ash from 100. The energy values were calculated by means of Atwater’s factors (protein and carbohydrate 4 kcal/g and fat 9 kcal/g, respectively). Chitin–chitosan of FPs was calculated as glucosamine equivalent after acid hydrolysis with hydrochloric acid (HCl). Briefly, 400 μL of 4 N HCl solution was added to freeze-dried and powdered FPs (10 mg) and incubated at 96 °C for 18 h. According to a previous method of Blix (1948), the glucosamine concentration of the supernatant was measured and taken as the amount of chitin-chitosan in FPs. β-glucan of FPs was measured by the mushroom and yeast beta-glucan assay kit (Megazyme international, Wicklow, Ireland).

### Mice and diet

All studies were carried out using 5-week-old male mice. C57BL/6N mice purchased from Japan SLC, Inc. (Shizuoka, Japan) and maintained at 22 °C in a humidity-controlled room at 55 ± 5% with a 12-h light-dark cycle. All mice were acclimatized for 1 week and randomly assigned to four groups based on the body weights, which were fed control AIN-93G diet (CO, *n* = 8), CO + 10% FPs (CO + FPs, *n* = 8), high-cholesterol diet including 0.50% cholesterol (Wako Pure Chemical Industries, Osaka, Japan) and 0.25% sodium cholate (HC, *n* = 7), and HC + 10% FPs (HC + FPs, *n* = 7). Based on the results of nutrient composition of FPs, the increased nutrient components by addition of FPs were, respectively, subtracted as follows: fat, soybean oil; carbohydrates, corn starch protein, casein; dietary fiber, cellulose. Indeed, the weight increased with addition of cholesterol and sodium cholate was subtracted from corn starch. Detailed diet compositions are provided in Table 1. The mice were fed the respective diets and provided with water ad libitum. The body weights and food intake were recorded for 8 weeks every other day and feces were collected as the same frequency as. To evaluate the time-periods during which the effects of FPs are detected, we analyzed the body weights of mice by dividing their feeding periods into middle and final body weight. Food intake and feeding efficiency calculated as follows: food intake (g food intake/day/100 g body weight) and feeding efficiency (g weight gain for 8 weeks/10 g food intake for 8 weeks). The feces were pooled for each individual and preserved every 2 weeks at −80 °C. Animal Experiment Committee of Miyazaki University approved this study (2013-024). The animal studies were conducted in accordance with the Guide for the Care and Use of Laboratory Animals of the University of Miyazaki and in compliance with the Law Concerning the Protection and Control of Animals (Japan Law No. 105), Standards Relating to the Care and Management of Laboratory Animals and Relief of Pain (Notification no. 88 of the Ministry of the Environment, Japan), and The Guidelines for Animals Experimentation (the Japanese Association for Laboratory Animal Science). After an overnight fast at the end of the feeding period, blood was collected from the heart under a triple anesthesia mix of 0.75 mg/kg medetomidine hydrochloride (Domitor; Nippon Zenyaku Kogyo Co., Ltd., Fukushima, Japan), 4.0 mg/kg midazolam (Dormicum; Astellas Pharma Inc., Tokyo, Japan), and 5.0 mg/kg butorphanol tartrate (Betorphal; Meiji Seika Pharma, Co., Ltd., Tokyo, Japan) and the mice were euthanized. Plasma was collected after centrifugation (2,000×*g* for 30 min at 4 °C) and stored at −80 °C until analysis. Epididymal/perirenal white adipose tissues and the livers were harvested, rinsed in saline, weighed, corrected by weight of each animal, frozen in liquid nitrogen, and stored at −80 °C.

**Table 1:** Composition of the experimental diet.

Component (g/kg)	CO	CO + FPs	HC	HC + FPs
Casein	200.00	159.37	200.00	159.37
Corn starch	397.50	385.58	390.00	378.08
Pregelatinized corn starch	132.00	132.00	132.00	132.00
Sucrose	100.00	100.00	100.00	100.00
Soybean oil	70.00	65.46	70.00	65.46
Cellulose	50.00	7.09	50.00	7.09
Vitamin mix^1^	10.00	10.00	10.00	10.00
Mineral mix^2^	35.00	35.00	35.00	35.00
Cystin	3.00	3.00	3.00	3.00
Choline bitartrate	2.50	2.50	2.50	2.50
*tertiary* Butylhydroquinone	0.01	0.01	0.01	0.01
Cholesterol	0.00	0.00	5.00	5.00
Sodium cholate	0.00	0.00	2.50	2.50
FPs	0.00	100.00	0.00	100.00
Total	1,000.01	1,000.01	1,000.01	1,000.01

**Notes:**

1 AIN-93G vitamin mixture.

2 AIN-93G mineral mixture.

FPs, fermented products prepared from sweet potato-*shochu* distillery by-product; CO, control diet; CO + FPs, control diet containing 10% FPs; HC, high-cholesterol diet including 0.50% cholesterol and 0.25% sodium cholate. HC + FPs: high-cholesterol diet containing 10% FPs.

### Biochemical analyses of plasma and liver samples

Plasma TG and TC levels, and aspartate aminotransferase (AST) and alanine transaminase (ALT) activities were measured using the TG *E*-test, cholesterol *E*-test and transaminase CII-test according to the manufacturer’s instructions (each from Wako), respectively.

The liver contents of TG and TC were measured after lipid extraction (Fujii et al., 2019). Briefly, liver tissue samples (100 mg) were taken in TM-626S tubes (TOMY SEIKO Co., Ltd., Tokyo, Japan). After adding four tablets of zirconia beads (Φ2.0) and 1 mL of phosphate-buffered saline to the tubes, the samples were homogenized (1,430×*g* for 3 min at 4 °C) by a beads cell disruptor Micro Smash MS-100R (TOMY SEIKO Co., Ltd., Tokyo, Japan). The homogenized cell suspension samples (800 μL) were transferred to test tubes containing 3.2 mL of chloroform-methanol (2:1) and mixed by vortexing for 2–3 min. After the samples were centrifuged (1,000×*g* for 1 min), the underlayers were collected into spitz tubes. The collected samples were dried using nitrogen gas in a water bath at 37 °C and dissolved with 2-propanol containing 5% (v/v) Tween-20.

### Western blotting

The liver tissue samples were homogenized in 50 mM Tris-HCl (pH 7.5), 150 mM NaCl, 1 mM EDTA, 50 mM NaF, 30 mM Na_4_P_2_O_7_, and 2% (v/v) Triton X-100 along with a protease inhibitor cocktail (Nacalai tesque, Inc., Kyoto, Japan). The protein concentration was determined using a BCA protein assay kit (Thermo Fisher Scientific, Inc., Waltham, MA, USA). Equal amounts of protein (20 μg) were loaded onto 4–12% NuPAGE Bis-Tris gels (Thermo Fisher Scientific, Inc., Waltham, MA, USA) and then transferred to PVDF membrane Power Blotter Select Transfer Stacks (Pore size: 0.2 μm, Thermo Fisher Scientific, Inc., Waltham, MA, USA) by electrophoretic transfer with 3-(*N*-morpholino) propanesulfonic acid running buffer (Thermo Fisher Scientific, Inc., Waltham, MA, USA). Subsequently, nonspecific binding sites were blocked with Blocking One-P (Nacalai tesque, Inc., Kyoto, Japan) for 30 min at 25 °C and the blots were incubated overnight at 4 °C. Next, the membrane was treated with antibodies against sterol regulatory element-binding protein-2 (SREBP-2, ab30682, 1:1,000), 3-hydroxy-3-methylglutaryl coenzyme A reductase (HMGR, ab174830, 1:1,000), liver X receptor α (LXRα, ab176323, 1:2,000), cholesterol 7 alpha-hydroxylase (Cyp7a1, ab65596, 1:1,000), β-actin (ab8226, 1:5,000) (all purchased from Abcam plc (Cambridge, UK)), and microtubule-associated protein 1 light chain 3 (LC3-I/-II, 12,741, 1:1,000) purchased from Cell Signaling Technology, Inc. (Danvers, MA, USA). After washing three times, the antigen-antibody complexes were visualized for 1 h at 25 °C with anti-rabbit IgG-HRP (Abcam plc, ab6721, 1:2,000) or anti-mouse IgG-HRP (Abcam plc, ab6789, 1:5,000). Signals were visualized using Pierce ECL western blotting substrate (Thermo Fisher Scientific, Inc., Waltham, MA, USA). Subsequently, bands were quantified using the chemiluminescent imaging system, WSE-6300 LuminoGraph III (ATTO Corporation, Tokyo, Japan). Expression values were obtained as relative expression levels of the target proteins (SREBP-2, HMGR, LC3-I/-II, LXR and Cyp7a1) normalized using a corresponding β-actin as an internal control with image analysis software CS Analyzer 4 (ATTO Corporation, Tokyo, Japan).

### Fecal cholesterol analysis

Freeze-dried feces were homogenized with dry ice using a laboratory crusher LAB MILL OML-1 (Osaka Chemical Co., Ltd., Osaka, Japan). Fecal cholesterol levels were analyzed by gas chromatography (GC) according to a previous report (Li et al., 2015). The crushed feces (100 mg) were transferred to 1.5 mL tubes and dissolved with 1 mL of methanol, followed by ultrasonic extraction for 30 min and centrifugation at 100×*g* for 10 min at 4 °C. The supernatants obtained (200 μL) were collected into spitz tubes and extracted with 1 mL of ethyl acetate after addition of 10 μL of an internal standard (100 μg/mL 5α-cholestane), 10 μL of methanol, and 500 μL of distilled water. Next, the samples were mixed using a vortex mixer for 10–20 min and centrifuged at 1,000×*g* for 10 min. The supernatants were dried under a nitrogen flow at 40 °C, and the residues were reconstituted with 200 μL of N, O-bis(trimethylsilyl)trifluoroacetamide containing 1% trimethylchlorosilane (Sigma-Aldrich, St. Louis, MO, USA) and were derivatized. One aliquot of each sample was used as a sample for GC analysis. The GC instrument consisted of a GC system (GC-2014; Shimadzu Corporation, Kyoto, Japan) and a capillary column SH-Rxi-5Sil MS (30 m × 0.25 mm i.d., thickness: 0.25 μm, Shimadzu Corporation, Kyoto, Japan). The parameters used were as follows: column temperature, 280 °C; injection temperature, 300 °C; detection temperature, 300 °C; carrier gas, helium (0.9 mL/min); injection mode, split (1:50); injection volume, 3.0 μL.

### Fecal bile acid analysis

Fecal bile acid (BA) components were analyzed by TechnoSuruga Laboratory Co. Ltd., (Shizuoka, Japan) using liquid chromatography in combination with hybrid quadrupole time-of-flight mass spectrometry (LC-QTOF-MS) according to a previous report (Kakiyama et al., 2014). The feces (100 mg) were transferred to bead tubes and dissolved with 0.9 mL of 50 mM sodium acetate buffer mixed with ethanol (1:3), followed by homogenization and heat treated for 30 min at 85 °C. After centrifugation (19,000×*g* for 10 min), the supernatant was diluted four times with water and applied to a Bond Elute C_18_ cartridge (Agilent Technologies Inc., Santa Clara, CA, USA). BAs were eluted with ethanol (5 mL). After the solvent was evaporated, the residue was dissolved in 1 mL of 50% ethanol. To an aliquot of this solution, 50% ethanol and internal standard (*d* 4-cholate and 23-nordeoxycholate, 1 μM in 50% ethanol) was added. Precipitated solids were removed by filtration through an Ultrafree-MC LG hydrophilic PTFE filter (UFC30LG00, 0.2 μm; Merck Millipore Ltd, Burlington, MA, USA). One aliquot of each sample was used as a sample for LC-QTOF-MS analysis. The LC-QTOF-MS instrument consisted of a Xevo G2-S QTOF (Waters Corporation, Milford, MA, USA) equipped with an electrospray ionization (ESI) probe and Waters ACQUITY UPLC systems (Waters Corporation, Milford, MA, USA). Injection volume was 4 μL. An Acquity UPLC BEH C18 column (150 × 2.1 mm id, 1.7 μm particle size; Waters Corporation, Milford, MA, USA) was employed at 60 °C. For the gradient analysis, the mobile phase A was water with 0.1% formate and the mobile phase B was acetonitrile with 0.1% formate. The separation was carried out by linear gradient elution at a flow rate of 0.5 mL/min. The gradient elution program of B was 25–35% for 0.5 min, 35–40% for 6.5 min, 40–50% for 4.0 min, 50–95% for 2.0 min, 95% for 1.0 min, 95–25% for 0.1 min and 25% for 4.9 min. The data acquisition was performed in negative ion ESI mode. The desolvation gas was nitrogen, and the collision gas was argon. The desolvation gas flow rate was 1,000 L/h. The source temperature was 150 °C and the desolvation temperature was 450 °C. The capillary voltage was 0.5 kV, cone voltage was 20.0 V. For mass accuracy, leucine-enkephalin was employed as the lock spray solution. The scan time for each function was set at 0.6 s. The data acquisition range was m/z 50–850. Next, we have used the TargetLynx Application Manager, an option with Waters MassLynx 4.1 software.

### SCFA analysis

Fecal SCFA levels were analyzed by TechnoSuruga Laboratory using GC according to a previous report (García-Villalba et al., 2012). The feces (100 mg) were transferred to bead tubes and suspended in 0.9 mL of water with 0.5% phosphoric acid, followed by heat treated for 30 min at 85 °C and centrifugation (19,000×*g* for 10 min). The supernatants obtained (400 μL) were collected into tubes. Each milliliter of the supernatant was extracted with the equal amount of ethyl acetate and centrifuged at 19,000×*g* for 10 min. Prior to analysis, a 200 μL volume of the ethyl acetate phase was transferred into a tube and an internal standard (1 mM 4-methyl valerate) containing 1% formate equivalent to the ethyl acetate phase added. One aliquot of each sample was used as a sample for GC analysis. The GC instrument consisted of a GC system (7890B; Agilent Technologies Inc., Santa Clara, CA, USA), a capillary column DB-WAXetr (30 m × 0.25 mm i.d., thickness: 0.25 μm, Agilent Technologies Inc., Santa Clara, CA, USA) and a guard column DB-WAXetr (5 m × 0.25 mm i.d., thickness: 0.25 μm, Agilent Technologies Inc., Santa Clara, CA, USA). The parameters used were as follows: column temperature, initially 50 °C, then increased to 90 °C at 10 °C/min, to 150 °C at 15 °C/min, to 170 °C at 5 °C/min, and finally to 250 °C at 20 °C/min and kept at this temperature for 4 min; injection temperature, initially 50 °C and finally to 250 °C at 250 °C/min; detection temperature, 250 °C; carrier gas, helium (1.2 mL/min); injection mode, pulsed splitless; injection volume, 1.0 μL.

### Gut microbiota analysis

Gut microbiota were analyzed by TechnoSuruga Laboratory. Fecal samples were collected and DNA extraction was performed according previous report (Takahashi et al., 2014). The variable V3–V4 region of the bacterial 16S rDNA was amplified by PCR (Takahashi et al., 2014). Sequencing was conducted using an Illumina Miseq sequencing system (Illumina, San Diego, CA, USA) with MiSeq Reagent Kit version 3 (600 cycle, Illumina, San Diego, CA, USA), according to the manufacturer’s instructions. Overlapping paired-end reads were merged using the fastq-join program (http://code.google.com/p/ea-utils/). Only reads that that had quality value scores of ≥20 for more than 99% of the sequence were extracted for further analysis. In addition, chimeric sequences were filtered out by UCHIME algorithm in USEARCH platform which performs reference based detection (USEARCH v6.1.544) (Edgar et al., 2011). The determined 16S rDNA sequences were subjected to homology searching using Metagenome@KIN software (World Fusion Co., Ltd., Tokyo, Japan) against the Ribosomal Database Project MultiClassifier ver. 2.11 (16S rDNA) (confidence: 0.8) for microorganisms identified at the phylum and the genus level (Wang et al., 2007). Furthermore, the relative abundance of each taxonomic classification was calculated from the classification and the number identified.

### Statistical analysis

All data are the means ± SE. All statistical analyses were performed using IBM SPSS Statistics version 24.0 (IBM Corporation, Armonk, NY, USA). Statistical analysis between two groups was performed employing an independent *t*-test. Analyses between four groups were performed by one-way analysis of variance with Tukey’s HSD post hoc test. A *p*-value < 0.05 was considered significant.

## Results

### Nutrient composition of FPs

Nutritive values of FPs per dry weight were investigated. FPs comprised 48.3% carbohydrate (37.8% dietary + 10.5% others), 35.8% proteins, 7.9% ash, 4.0% fat, 4.0% moisture and 297.0 kcal/100 g. Similar to previous reports (Kosakai et al., 2019), FPs were rich in dietary fiber such as 16.7% β-glucan and 12.4% chitin-chitosan.

### Effect of FPs on growth parameters in mice

There was no significant difference observed between the middle body weights of the CO group and the CO + FPs group; however, final body weights in the CO + FPs group were significantly lower than those in the CO group (Table 2). On the contrary, both the middle and final body weights of the HC + FPs group were significantly lower than that of the HC group, and the effects on body weights were detected earlier in cholesterol loading conditions (Table 2). In order to evaluate the relationship between food intake and suppression of weight gain, food intake, and feeding efficiency were confirmed. Food intake of the groups that were fed diets containing FPs (CO + FPs and HC + FPs groups) were not significantly changed compared with those of the respective control groups (CO and HC groups). There was no significant difference in feeding efficiency between the CO and the CO + FPs groups, however, indicating that FPs significantly lowered feeding efficiency in the cholesterol loaded conditions (Table 2).

**Table 2:** Effect of FPs on growth parameters in mice.

	CO	CO + FPs	HC	HC + FPs
Body weight (g)				
Initial	20.88 ± 0.32^a^	20.91 ± 0.31^a^	20.68 ± 0.26^a^	20.80 ± 0.24^a^
Middle	27.12 ± 0.69^a^	25.44 ± 0.35^a^	25.46 ± 0.39^a^	23.40 ± 0.05^b^
Final	32.70 ± 0.73^a^	29.62 ± 0.75^b^	28.81 ± 0.65^b^	24.31 ± 0.27^c^
Food intake (g/day/100 g-body weight)	11.09 ± 0.46^a^	10.95 ± 0.31^a^	13.06 ± 0.44^b^	12.67 ± 0.25^b^
Feeding efficiency (g/10 g)	0.59 ± 0.05^a^	0.48 ± 0.04^a^^,^^b^	0.39 ± 0.03^b^	0.20 ± 0.02^c^
Liver weight (g/100 g-body weight)	3.51 ± 0.08^a^	3.61 ± 0.04^a^^,^^b^	4.09 ± 0.04^b^	7.22 ± 0.76^c^
Perirenal adipose weight (g/100 g-body weight)	1.95 ± 0.17^a^	1.39 ± 0.15^a^^,^^b^	1.04 ± 0.15^b^	0.28 ± 0.04^c^
Epididymal adipose weight (g/100 g-body weight)	4.26 ± 0.26^a^	3.27 ± 0.32^a^^,^^b^	2.92 ± 0.33^b^	1.01 ± 0.09^c^
Fecal wet weight (g/day)	0.29 ± 0.01^a^	0.33 ± 0.01^b^^,^^c^	0.32 ± 0.01^a^^,^^b^	0.36 ± 0.01^c^

**Notes:**

Values represent mean ± SE, *n* = 7–8. Means in a row followed by differing superscript letters (a, b, c) indicate statistically significant difference, *p* < 0.05.

FPs, fermented products prepared from sweet potato-*shochu* distillery by-product; CO, control diet; CO + FPs, control diet containing 10% FPs; HC, high-cholesterol diet including 0.50% cholesterol and 0.25% sodium cholate; HC + FPs, high-cholesterol diet containing 10% FPs; Initial, body weight on Day0; Middle, body weight on Day28; Final, body weight on Day56. Fecal wet weight was measured using feces pooled from the 1st to 8th week.

Regarding tissue weights, the weight of liver was not significantly different between the CO group and the CO + FPs group, and was significantly increased in the HC + FPs group compared with those of the HC groups (Table 2). On the contrary, the weight of perirenal and epididymal adipose was significantly reduced in the HC + FPs group compared with that in the HC groups, and that in the CO + FPs group did not.

### Effect of FPs on biochemical parameters in serum and liver

We analyzed the mice serum as shown in Table 3. The serum TC levels in the CO + FPs group were significantly lower than those in the CO group; conversely the serum TC levels of the HC + FPs group were significantly higher than those of the HC group (Table 3). Especially, FPs significantly increased the serum non-HDL-C levels, and the ratio of non-HDL to HDL was also higher in mice fed cholesterol-loaded diet compared with that of the HC group (Table 3). Also, in the liver, the TC levels were significantly increased only in the HC + FPs group (Table 3). However, serum TG levels were not significantly different between the CO group and the CO + FPs group, and were significantly reduced in the HC + FPs group compared with those of the HC groups (Table 3). Furthermore, the TG levels in the livers of the CO + FP and the HC + FPs group were significantly decreased than those in the livers of the CO and HC groups, respectively (Table 3). The serum AST and ALT activities were significantly higher in the HC + FPs group than those in the other groups (Table 3). There were no significant differences in serum total BA levels and total protein concentration between all the groups (Table 3).

**Table 3:** Effect of FPs on serum, hepatic and fecal biochemical parameters in mice fed with different experimental diet.

	CO	CO + FPs	HC	HC + FPs
Serum				
TC (mg/dL)	123.76 ± 4.66^a^	102.76 ± 3.89^b^	99.93 ± 3.98^b^	159.43 ± 7.95^c^
HDL-C (mg/dL)	77.11 ± 8.80^a^	68.76 ± 3.15^a^^,^^b^	55.34 ± 2.56^b^^,^^c^	38.91 ± 0.73^c^
non-HDL-C (mg/dL)	46.65 ± 4.66^a^	33.99 ± 1.65^a^	45.59 ± 3.12^a^	120.52 ± 8.02^b^
non-HDL-C/ HDL-C	0.69 ± 0.13^a^	0.50 ± 0.03^a^	0.82 ± 0.07^a^	3.11 ± 0.22^b^
TG (mg/dL)	85.21 ± 7.58^a^	88.88 ± 3.04^a^	58.81 ± 4.87^b^	33.80 ± 1.65^c^
total BA (μmol/L)	7.24 ± 4.24^a^	7.97 ± 3.13^a^	4.73 ± 1.86^a^	11.22 ± 2.97^a^
AST (U/L)	63.62 ± 8.37^a^	96.45 ± 16.72^a^^,^^b^	164.67 ± 45.57^a^^,^^b^	192.09 ± 38.05^b^
ALT (U/L)	27.45 ± 3.99^a^	21.73 ± 4.20^a^	20.99 ± 3.50^a^	139.23 ± 27.17^b^
Total protein (mg/L)	45.07 ± 0.61^a^	46.81 ± 1.38^a^	44.33 ± 1.13^a^	47.45 ± 0.73^a^
Liver				
TC (mg/g liver)	5.48 ± 0.36^a^	4.13 ± 0.09^a^	29.51 ± 2.92^b^	103.59 ± 8.01^c^
TG (mg/g liver)	99.68 ± 13.91^a^	62.16 ± 4.51^b^^,^^c^	84.82 ± 8.29^a^^,^^b^	43.23 ± 4.28^c^
Feces				
TC (mg/g feces)	17.88 ± 0.89^a^	16.05 ± 0.76^a^	87.02 ± 5.83^b^	250.87 ± 13.68^c^

**Notes:**

Values represent mean ± SE, *n* = 7–8. Means in a row followed by differing superscript letters (a, b, c) indicate statistically significant difference, *p* < 0.05.

FPs, fermented products prepared from sweet potato-*shochu* distillery by-product; CO, control diet; CO + FPs, control diet containing 10% FPs; HC, high-cholesterol diet including 0.50% cholesterol and 0.25% sodium cholate; HC + FPs, high-cholesterol diet containing 10% FPs; TC, total cholesterol; HDL-C, high-density lipoprotein cholesterol; non-HDL-C, non-high-density lipoprotein cholesterol; TG, triglyceride; BA, bile acids; AST, aspartate transaminase; ALT, alanine transaminase. Fecal TC was measured using feces pooled from the 7th to 8th week.

### Effect of FPs on the expression of cholesterol- and bile acids-related proteins in the liver

**Figure 1:**
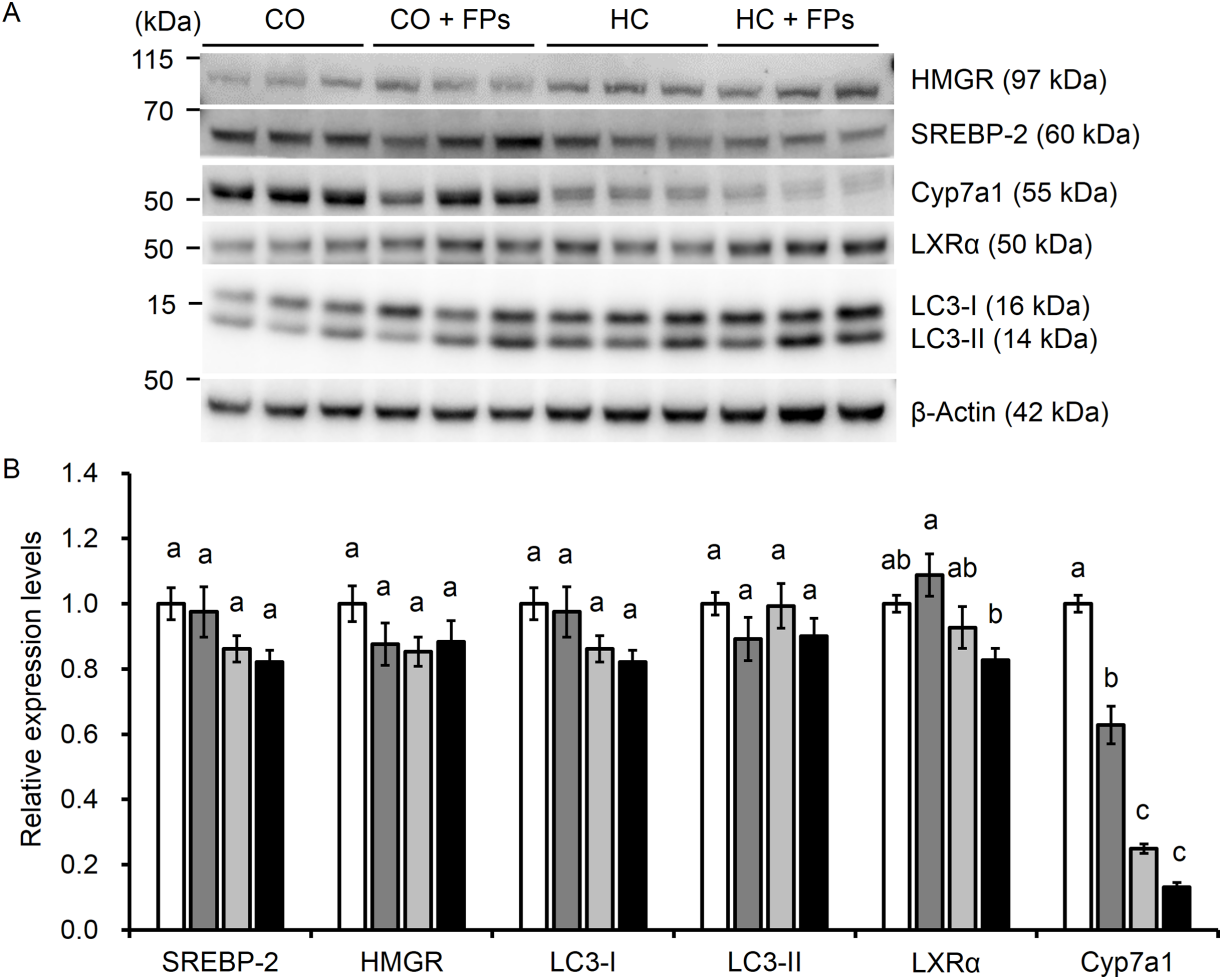
Effect of FPs on the expression of proteins involved in cholesterol and bile acid metabolism in the liver. (A) The intensity of the bands was quantified by densitometric analysis and normalized with corresponding bands of β-actin. (B) Relative expression levels of each proteins. Values represent mean ± SE, *n* = 7–8. Means in a row followed by differing superscript letters (a, b, c) indicate statistically significant difference, *p* < 0.05. FPs, fermented products prepared from sweet potato-*shochu* distillery by-product; CO, control diet, CO + FPs, control diet containing 10% FPs; HC, high-cholesterol diet including 0.50% cholesterol and 0.25% sodium cholate; HC + FPs, high-cholesterol diet containing 10% FPs; SREBP-2, sterol regulatory element-binding protein-2; HMGR, 3-hydroxy-3-methylglutaryl coenzyme A reductase; LC3, microtubule-associated protein 1 light chain 3, LXRα, liver X receptor α; Cyp7a1, cholesterol 7 alpha-hydroxylase.

Fermented products did not affect mature SREBP-2, HMGR, LC3-I and -II protein expressions (Fig. 1A). The relative expression levels of LXRα in the HC + FPs group were significantly decreased than in the CO + FPs group, however, there were no significant differences with the addition of FPs than without. The expression levels of Cyp7a1 in the CO + FPs group were significantly lower than those in the CO groups, however, there were no significant differences between in the HC and the HC + FPs group (Figs. 1A and 1B).

### Effect of FPs on cholesterol and bile acid excretion into feces

As shown in Table 2, fecal wet weight of the groups that were fed diets containing FPs (CO + FPs and HC + FPs groups) were significantly increased compared with those of the respective control groups (CO and HC groups). The results of the feces pooled from the 7th to 8th week of feeding indicated that the fecal TC levels were not significantly different between the CO and the CO + FPs groups; however, these levels significantly increased in the HC + FPs groups compared with those in the HC group (Table 3). When the BA contents in feces pooled on the day before euthanize were analyzed for the two groups loaded with cholesterol (HC and HC + FPs groups), there were no significant differences between these groups (Table 4). Cholate, which is the primary BA, was significantly increased by FPs administration, but chenodeoxycholate and α-murycholate were significantly decreased; however, there was no significant difference in β-muricholate levels between the two groups (Table 4). In addition, the secondary BAs such as deoxycholate, lithocholate, ursodeoxycholate, and hyodeoxycholate were significantly reduced (Table 4), but there was no significant difference in taurodeoxycholate and ω-muricolate levels between the two groups. Besides, when the results of BAs composition were separately analyzed from primary and secondary BA, no significant difference was found in primary BA the two groups, but secondary BA was significantly reduced by the addition of FPs (Table 4).

**Table 4:** Effect of FPs on bile acid profiles in feces.

BA (μmol/g feces)	HC	HC + FPs
Cholate	2.46 ± 0.51	5.64 ± 0.79*
Deoxycholate	10.75 ± 0.97	5.63 ± 0.82*
Chenodeoxycholate	0.08 ± 0.01	0.05 ± 0.00*
Lithocholate	0.17 ± 0.01	0.03 ± 0.01*
Ursodeoxycholate	0.06 ± 0.01	0.01 ± 0.01*
Hyodeoxycholate	0.07 ± 0.03	0.00 ± 0.00*
Glycocholate	N.D.	N.D.
Taurocholate	0.16 ± 0.07	0.99 ± 0.48
Taurodeoxycholate	0.16 ± 0.04	0.41 ± 0.18
Tauro-β-muricholate	0.16 ± 0.10	0.41 ± 0.27
α-Muricholate	1.26 ± 0.14	0.27 ± 0.02*
β-Muricholate	3.62 ± 0.69	2.20 ± 0.06
ω-Muricholate	1.59 ± 0.64	0.19 ± 0.11
Total BA	20.83 ± 1.65	15.83 ± 1.40
Primary BA	7.41 ± 1.16	8.16 ± 0.78
Secondary BA	12.64 ± 0.83	5.86 ± 0.93*

**Notes:**

Values represent mean ± SE, *n* = 4. Asterisk (*) indicates statistically significant differences compared with HC groups, *p* < 0.05.

FPs, fermented products prepared from sweet potato-*shochu* distillery by-product; HC, high-cholesterol diet including 0.50% cholesterol and 0.25% sodium cholate; HC + FPs, high-cholesterol diet containing 10% FPs; BA, bile acids; N.D., not detected. Primary BA was calculated as the sum of cholate, chenodeoxycholate, α-muricholate and β-muricholate, and Secondary BA was calculated as the sum of deoxycholate, lithocholate, ursodeoxycholate, hyodeoxycholate, and ω-muricholate

### SCFAs in feces

Analysis of fecal SCFAs showed that total SCFAs and propionate levels significantly increased in the HC + FPs group than in the CO group, however, there were no significant differences in the CO + FPs and the HC + FPs diet groups compared to their respective control groups (Table 5). No significant differences were observed between each groups in fecal acetate (Table 5). *n*-Butyrate was significantly increased in the CO + FPs and the HC + FPs groups compared to their respective control groups; furthermore, the *n*-butyrate levels in the HC + FPs group were significantly higher than those in the CO + FPs group (Table 5).

**Table 5:** SCFA content in the feces of mice.

	CO	CO + FPs	HC	HC + FPs
Total SCFAs (μmol/g feces)	3.92 ± 0.47^a^	10.53 ± 2.21^a^^,^^b^	6.53 ± 0.73^a^^,^^b^	13.29 ± 3.58^b^
Propionate (μmol/g feces)	0.00 ± 0.00^a^	0.60 ± 0.35^a^^,^^b^	0.41 ± 0.24^a^^,^^b^	3.46 ± 1.42^b^
Acetate (μmol/g feces)	3.82 ± 0.47^a^	8.51 ± 1.74^a^	5.63 ± 0.48^a^	7.47 ± 1.94^a^
*n*-Butyrate (μmol/g feces)	0.00 ± 0.00^a^	1.27 ± 0.11^b^	0.00 ± 0.00^a^	1.71 ± 0.16^c^

**Notes:**

Values represents mean ± SE, n = 4. Means in a row followed by differing superscript letters (a, b, c) indicate statistically significant difference, *p* < 0.05. Total SCFAs were calculated as the sum of propionate, acetate, *n*-butyrate, *iso*-butyrate, *n*-valerate, *iso*-valerate and *n*-capronate.

SCFA, short chain fatty acid; FPs, fermented products prepared from sweet potato-*shochu* distillery by-product; CO, control diet; CO + FPs, control diet containing 10% FPs; HC, high-cholesterol diet including 0.50% cholesterol and 0.25% sodium cholate; HC + FPs, high-cholesterol diet containing 10% FPs.

### Gut microbiota

**Figure 2:**
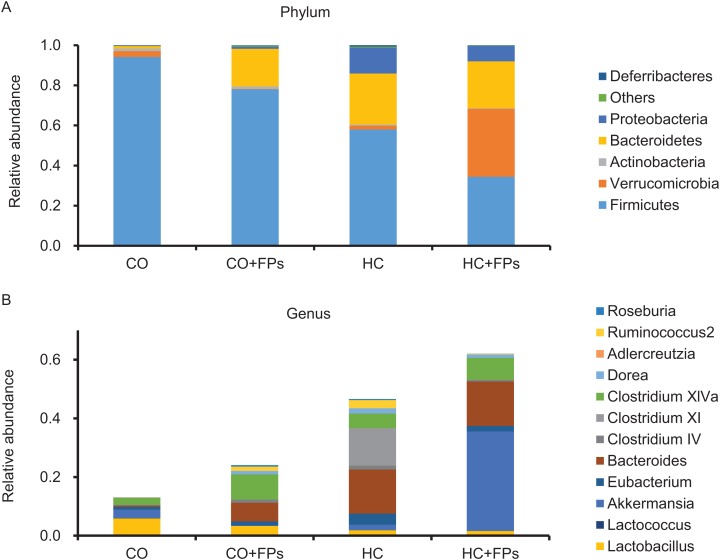
Modulation of gut microbiota by FPs. Fecal microbiota composition of mice after 8 weeks on each diet. CO, control diet; CO + FPs, control diet containing 10% FPs; HC, high-cholesterol diet including 0.50% cholesterol and 0.25% sodium cholate; HC + FPs, high-cholesterol diet containing 10% FPs; FPs, fermented products prepared from sweet potato-*shochu* distillery by-product. Relative abundance at the bacterial (A) *phylum* level and (B) *genus* level in the taxonomic contribution of fecal microbial communities.

The effects of FPs on intestinal environment were evaluated by analysis of the intestinal flora using pooled feces in the different groups one day before the mice were euthanized. At the *phylum* level, FPs reduced the abundance of *Firmicutes* (Fig. 2A). The abundance of *Bacteroidetes* increased with the addition of FPs only in the normal diet group, but did not increase in the cholesterol-loaded diet group (Fig. 2A). The number of *Verrucomicrobia* increased only in the HC + FPs group compared with those of the other groups (Fig. 2A). Detailed analysis at the *genus* level showed that the abundance of *Clostridium* cluster IV did not change, while that *Clostridium* cluster XI was significantly higher only in the HC group (composition ratio CO, CO + FPs, and HC + FPs: 0.0%, HC: 12.8%). *Clostridium* cluster XIVa increased in the CO + FPs and the HC + FPs groups (1.5-fold in the HC + FPs group compared with that of the HC group) (Fig. 2B). In loading FPs, the abundance of *Clostridium* cluster IV and XIVa increased 3.7-fold in the CO + FPs group and increased 1.3-fold in the HC + FPs group compared to their respective control groups, respectively. Besides, *Akkermansia* significantly increased only in the HC + FPs groups compared with that of the other groups (Fig. 2B).

## Discussion

In this study, we reported that FPs were rich in dietary fiber, such as β-glucan and chitin-chitosan (Kosakai et al., 2019). Consequently, CO + FPs and HC + FPs diet contained 1.7% β-glucan and 1.2% chitin-chitosan. Therefore, it is reasonable to attribute the body-fat lowering effect of FPs to these dietary fibers. Our results are similar to those of Miyazawa et al. (2018) who reported that ICR mice fed a diet supplemented with 1.0% chitosan for 6 weeks showed decreased body weight compared with mice fed high-fat diet. It has been reported that dietary chitosan-suppressed weight gain was apparent from the 5th week onward, wherein chitosan prevented intestinal absorption of lipids (Han et al., 2005; Sumiyoshi & Kimura, 2006). Our data agree with this report, in that 5 weeks were required to exert the inhibitory effects of FPs on weight gain. Therefore, at least in part, it is reasonable to explain the suppression of weight gain, which is caused by inhibiting lipid absorption irrespective of cholesterol intake. We also reported that FPs reduced weight gain in normal diet conditions (Kosakai et al., 2019) and also led to a similar decrease in cholesterol-loaded conditions. Therefore, it can be suggested that dietary FPs lowered weight gain by decreasing the weight of adipose tissues in mice fed not only a normal diet but also high-cholesterol diet.

It was indicated that the effect of dietary cholesterol in this animal strain tended to appear in the liver and to be less responsive to the serum parameter (Sehayek et al., 2000), being reasonable for our study. It is reported that dietary chitosan (Liu et al., 2018), and 1,3- or 1,3-1,6-β-glucan derived from yeast (Vetvicka & Vetvickova, 2009) and fungus (Rop, Mlcek & Jurikova, 2009) prevent elevation of serum TC levels in cholesterol-fed animals. Hence, we hypothesized that FPs, being rich in β-glucan and chitin-chitosan, reduce serum TC levels in cholesterol-loaded conditions. It has become necessary to investigate the effects of FPs using modified mice with cholesterol metabolism similar to humans (Hartvigsen et al., 2007; Chan, 2015). Partially, the increased serum TC levels in our study suggest that there are components that offset the effects of dietary fiber and further raise the cholesterol pool. Besides, the raised serum ALT levels indicated the induction of liver injury. It has been reported that dietary chitosan (Liu et al., 2018) and reishi extract (Meneses et al., 2016) cancelled the raised serum AST and ALT levels; however, these results differ from those observed in our studies. Besides, our studies indicated that dietary FPs decreased the hepatic and serum TG levels in cholesterol-loaded conditions. This observation is convincing for us because reishi extract, which is rich in 1,3-β-glucan (Meneses et al., 2016) and chitosan (Miyazawa et al., 2018; Liu et al., 2018) reduced hepatic and serum TG levels, and both are representative fibers present in the FPs of our study.

Hepatic cholesterol levels are strictly maintained through manipulation of the activity of transcriptional factor, SREBP-2, resulting in the modulation of HMGR expression which is a rate-limiting enzyme for cholesterol synthesis. It has been reported that the expression of SREBP-2 and LC3-II, an autophagy-related protein, are negatively correlated with each other (Kim & Guan, 2011; Eid et al., 2017). Furthermore, LXRα is a nuclear receptor and regulates the expression of Cyp7a1, which is a rate-limiting enzyme for BA synthesis. Although a previous study indicated that an intake of 5% chitosan increases the activities of hepatic Cyp7a1 in Sprague-Dawley rats (Moon et al., 2007), FPs decreased its protein expression instead of showing an increase. Therefore, cholesterol accumulation in FPs group may be explained by insufficient catabolism from cholesterol to BA. To interpret the present data, information on the type of mouse strains used may be critical factor. It has been reported that cholesterol- and BA metabolism- associated protein expressions do not change by dietary cholesterol load in some mice strains (Khanuja et al., 1995; Lammert et al., 1999). Among the measured proteins, only Cyp7a1 but not others were decreased under dietary cholesterol-loading as also shown in a previous study (Xu et al., 2004). It is important to accumulate information of the effects of FPs on other strains or models to predict their effects on human cholesterol metabolism. We also focused on the excretion of BA and its composition, because chitosan has been reported to bind with BAs and increase their excretion in feces, which is found to be higher than that with cellulose and glucomannan (Gallaher et al., 2000). However, FPs failed to promote BA excretion. The protein expression levels of Cyp7a1 tended to decrease in the HC + FPs group than in the HC group (Fig. 1). As the result, the BA excretion from the liver may be reduced, and BA absorption from the intestinal tract may have been promoted. It was reported that fecal lipid excretion was increased by dietary chitosan and/or glucomannan than that by cellulose in Wistar rats (Gallaher et al., 2000), and water-soluble chitosan bound to cholesterol rather than cellulose (Jin et al., 2017). Therefore, it is reasonable to consider that dietary fiber contained in FPs inhibits lipid absorption and promotes its excretion into feces. In contrast, cholesterol is excreted into the intestinal tract via ABCG5/ABCG8 (Lu, Lee & Patel, 2001). Further studies are needed to clarify the effect of FPs on intestinal ABC transporter function.

Intriguingly, FPs decreased secondary BA excretion; such BAs have been reported to cause DNA damage through ROS production (Ridlon, Kang & Hylemon, 2006; Payne et al., 2007), cell senescence (Friedman, 2008), and liver cancer (Yoshimoto et al., 2013). Several reports revealed that *Bacteroides* species (*Clostridium* cluster XI and XIVa and *Bacteroidetes fragilis*) (Hirano & Masuda, 1982; Brook, 1989; Ridlon et al., 2014; Fiorucci & Distrutti, 2015) are responsible for the dehydrogenation of primary bile, resulting in secondary BA production. Here, *Clostridium* cluster XI and XIVa were 0.4 times the cumulative amount in the HC + FPs group compared with those in the HC groups, suggesting that gut microbiota that contributed to secondary BA production were present. Changes of the abundance of *Clostridium* cluster XI and XIVa at the *genus* level were also in agreement with the results of laminarin (Nguyen et al., 2016), suggesting the effect of dietary fiber intake of FPs.

Gut microbiota produces SCFAs and provides benefits to the host (Lattimer & Haub, 2010). Among them, propionate has an inhibitory effect on cholesterol synthesis (Ide, Okamatsu & Sugano, 1978; Amaral et al., 1992; Lattimer & Haub, 2010). Although FPs promoted propionate level in the feces, FPs failed to inhibit cholesterol accumulation and the protein expression involved in cholesterol synthesis in mice. Therefore, it is considered that upregulation of propionate is not enough to downregulate cholesterol synthesis as indicated in our study. It has been reported that *Clostridium* cluster IV and XIVa, which are the main component strains of the *genus Firmicutes*, produce butyrate and greatly contribute to maintain the intestinal immune homeostasis (Atarashi et al., 2013). In our study, dietary FPs increased the abundance of *Clostridium* cluster IV and XIVa, suggesting that they partially contributed to increase butyrate production. Among the *genus Akkermansia*, *Akkermansia muciniphila* was the only intestinal bacterium reported to be present; it has been reported to produce acetate and propionate using mucin (Derrien et al., 2004). It was suggested that intestinal bacteria that convert acetate to butyrate are one of the causes of increasing SCFA levels in feces. Since butyrate suppresses inflammation (Vinolo et al., 2011) and has anti-cancer activity (Canani et al., 2011), FPs might be a putative candidate for the inhibition of intestinal inflammation caused by upregulation of SCFAs with subsequent reduction of secondary BAs.

It has also been reported that some intestinal bacteria control host energy metabolism (Backhed et al., 2004; Velagapudi et al., 2010; Den Besten et al., 2013). More *Firmicutes* and less *Bacteroidetes* (Turnbaugh et al., 2006) are reported to be present in obese mice, and *Bacteroidetes* increase and *Firmicutes* decrease in mice fed pectin (Trompette et al., 2014) and laminarin (β-1, 3-glucan) (Nguyen et al., 2016). In our study, the consistency between the suppression of weight gain and modulation of gut microbiota at the *phylum* level may be suggested to be due to dietary fiber. *Akkermansia* has been shown to be associated with high-fat diets (Jakobsdottir et al., 2013; Zhong et al., 2015). In contrast, there are reports related to anti-obesity (Everard et al., 2013) activities of *Akkermansia*, attracting attention as a useful next-generation microorganism (Cani & De Vos, 2017; Naito, Uchiyama & Takagi, 2018). Further analysis is necessary to investigate the significance of the increased abundance of *Akkermansia* in this study.

In our study, it is demonstrated that the FPs ingestion significantly reduced fat accumulation but increased the liver and serum cholesterol levels in high-cholesterol diet groups. Furthermore, it is suggested that this action is due to components other than dietary fiber in the diet. Since the effects of FPs change depending on the food composition, we would like to explore conditions that exhibit maximum effects in the future. In addition, it is also suggested that dietary FPs decreased secondary BA and increased SCFA by modulating gut microbiota.

## Conclusions

Dietary FPs decreased hepatic and serum TG levels in cholesterol-loaded conditions, while contrary to our hypothesis increasing hepatic and serum TC levels in cholesterol loaded mice, thus suggesting that there are components which offset the effects of dietary fiber in FPs. Moreover, alterations of the gut microbiota accompanied with reduction of secondary BAs and increase of SCFAs were observed in mice fed dietary FPs, which indicated that dietary fiber present in FPs might be responsible for this effect. Further studies are needed to reveal the mechanisms to increase the cholesterol pool by FPs for its utilization as a beneficial food source with anti-obesity effects.

## Supplemental Information

10.7717/peerj.7671/supp-1 Supplemental Information 1 Raw data.

## Additional Information and Declarations

### Data Availability

The following information was supplied regarding data availability: The raw measurements are available in the Supplemental File.
