# Supplementary figures and images for: Dietary fermented products using koji mold and sweet potato-shochu distillery by-product promotes hepatic and serum cholesterol levels and modulates gut microbiota in mice fed a high-cholesterol diet
Source: PeerJ. 2019 Sep 12;7:e7671. doi: 10.7717/peerj.7671 (PMC6745188; doi:10.7717/peerj.7671)

SREBP-2

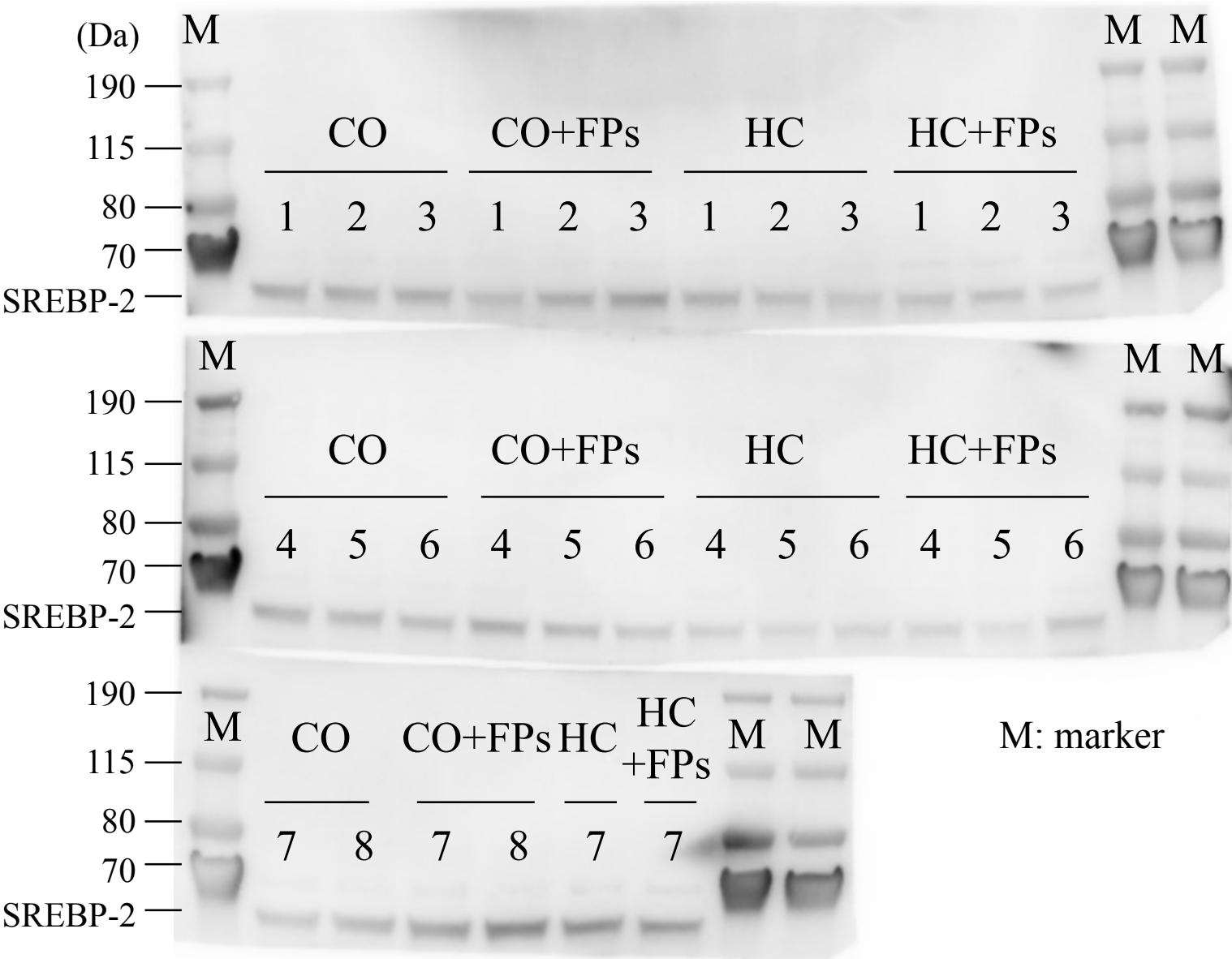

Cyp7a1

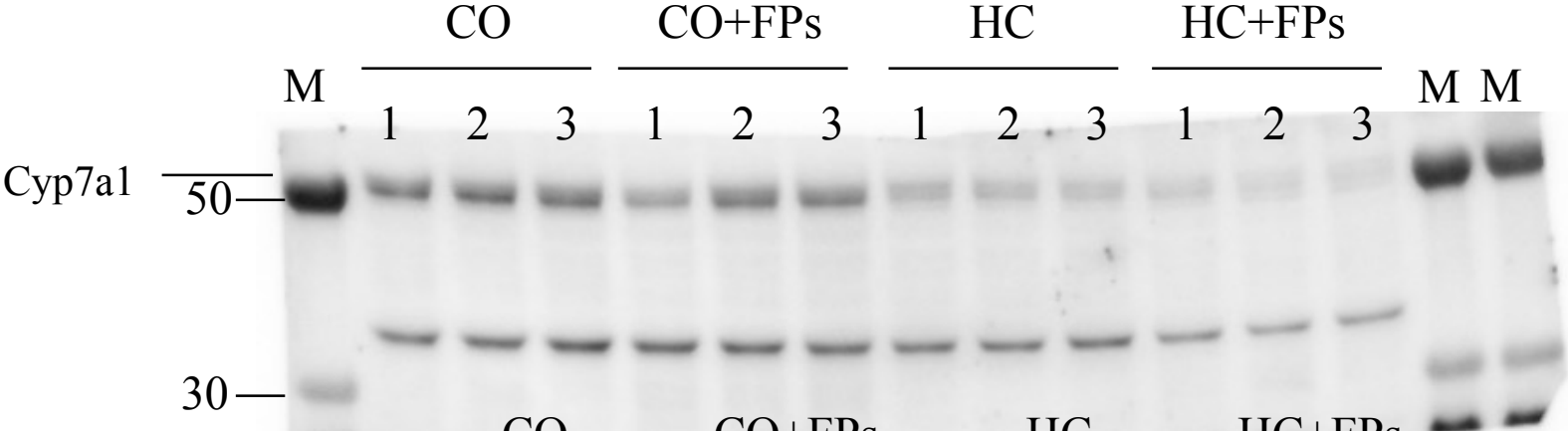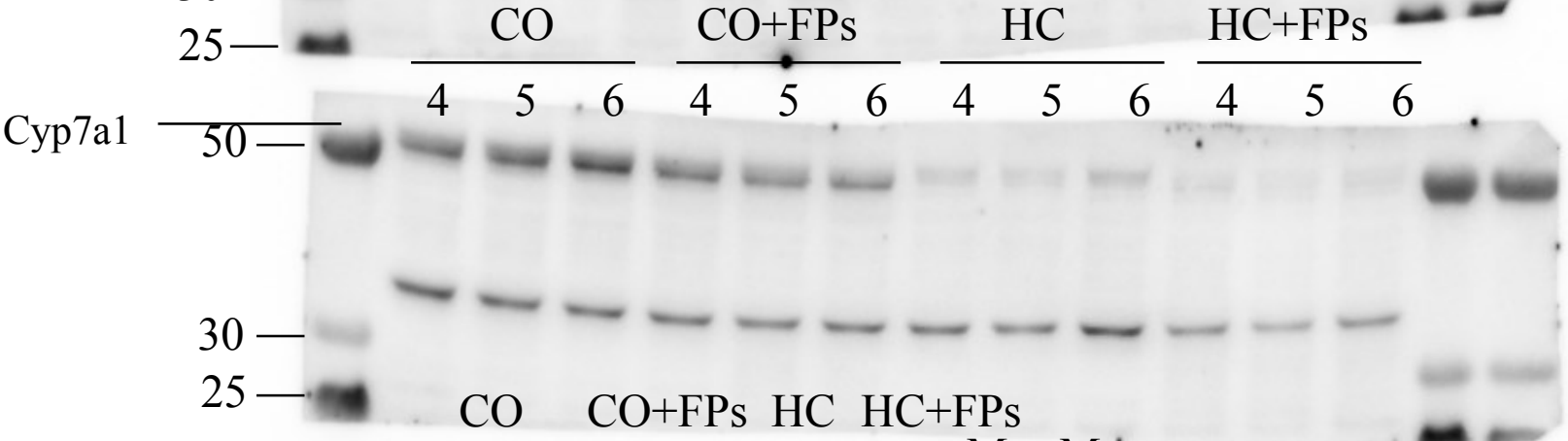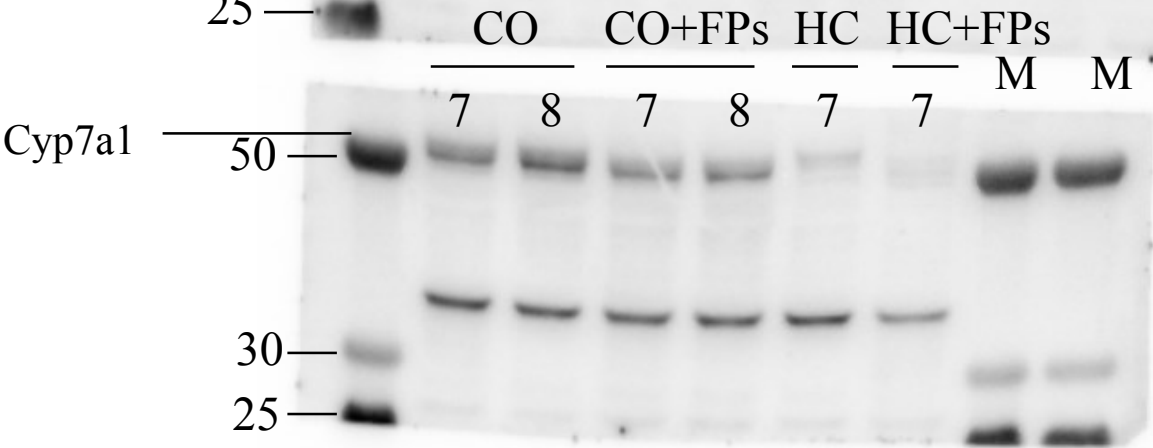

(kDa)

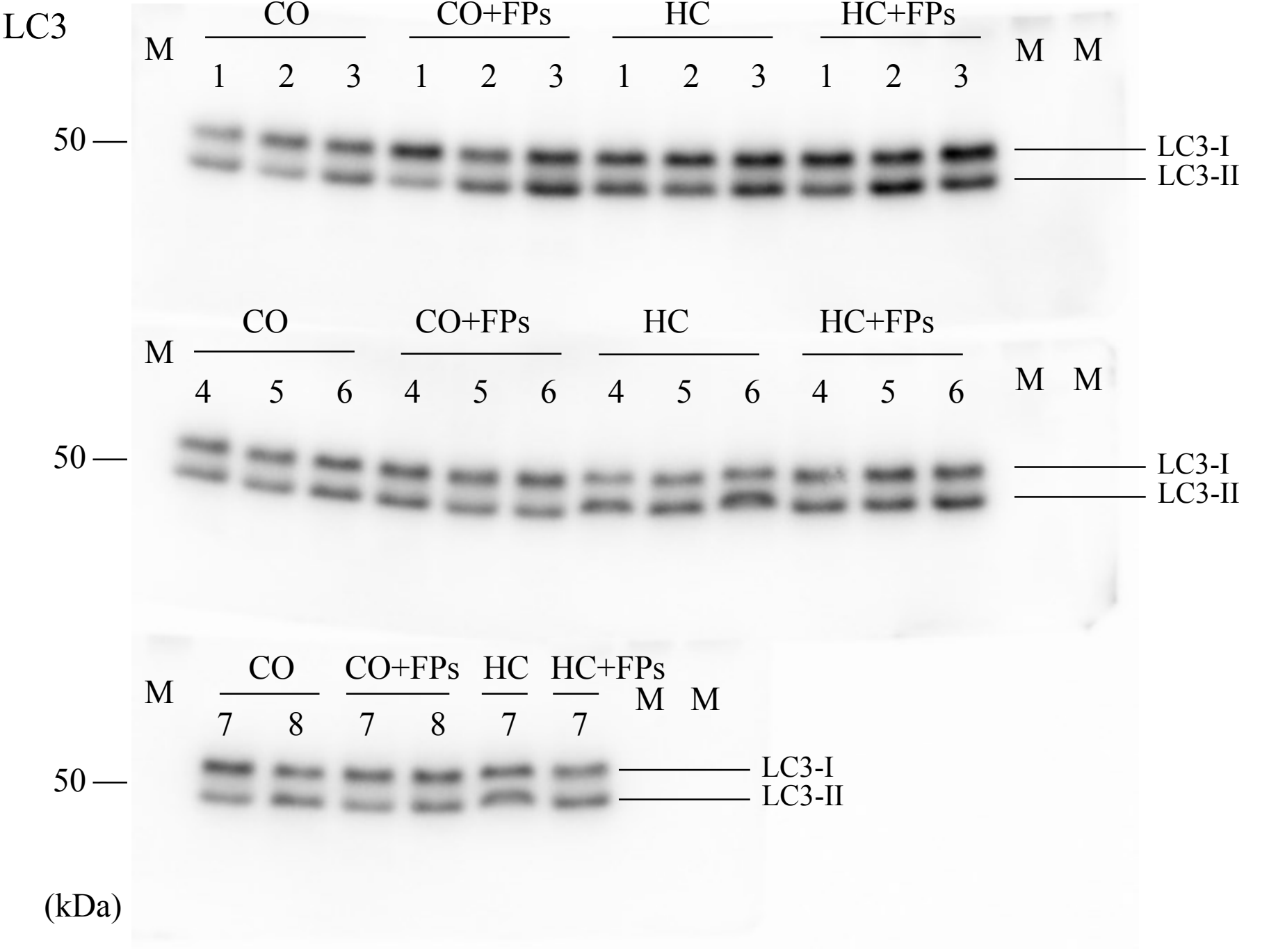

HMGR

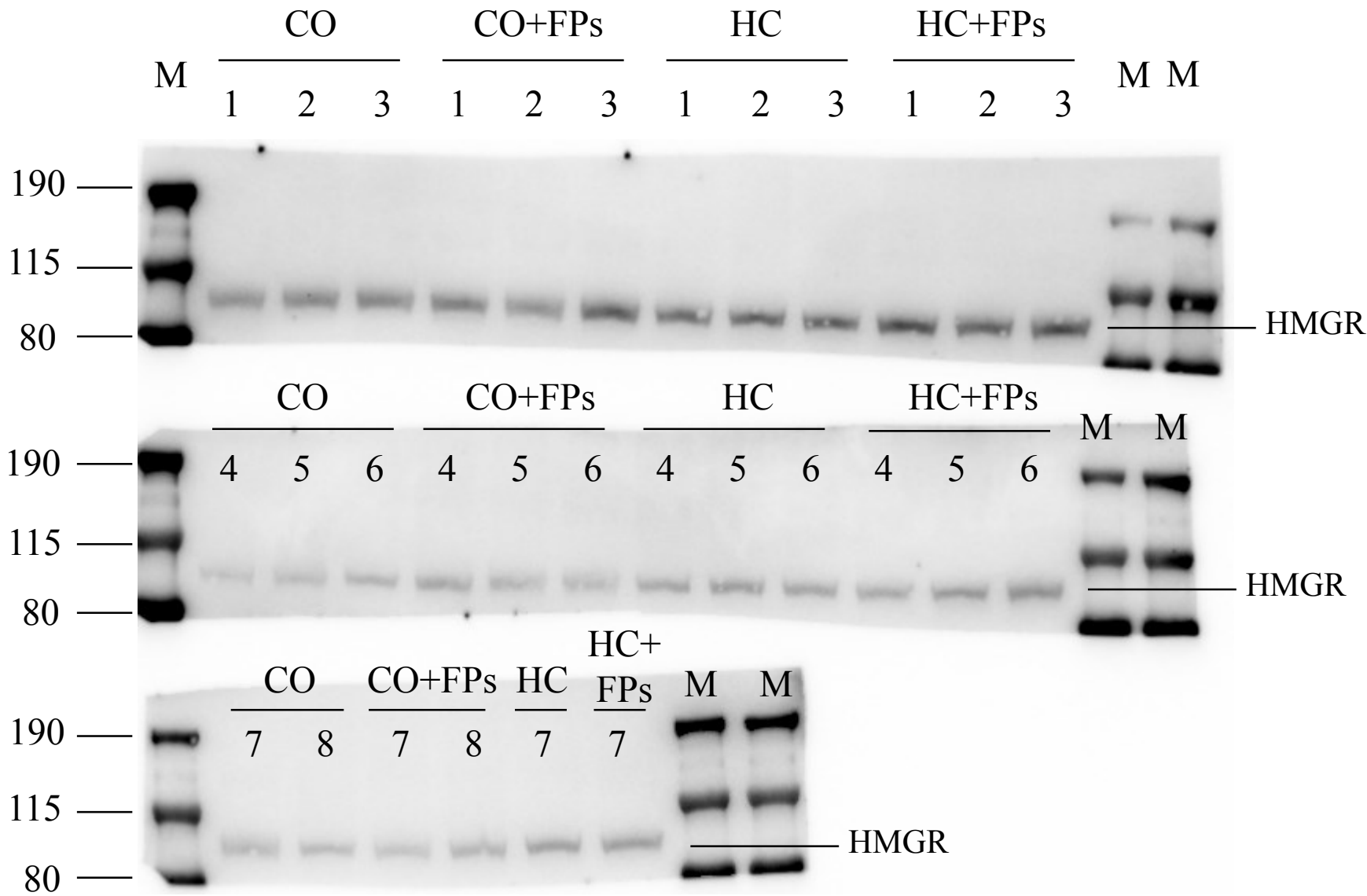

(kDa)

LXR $\alpha$

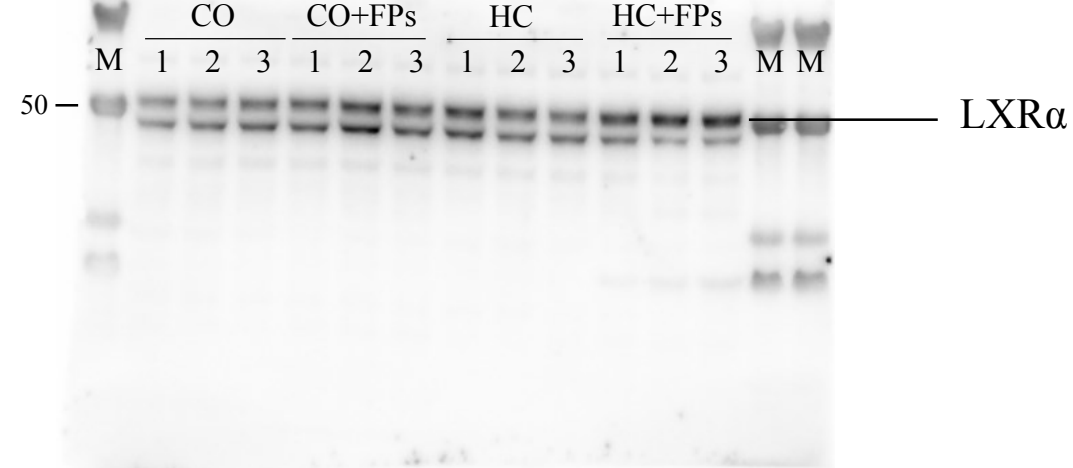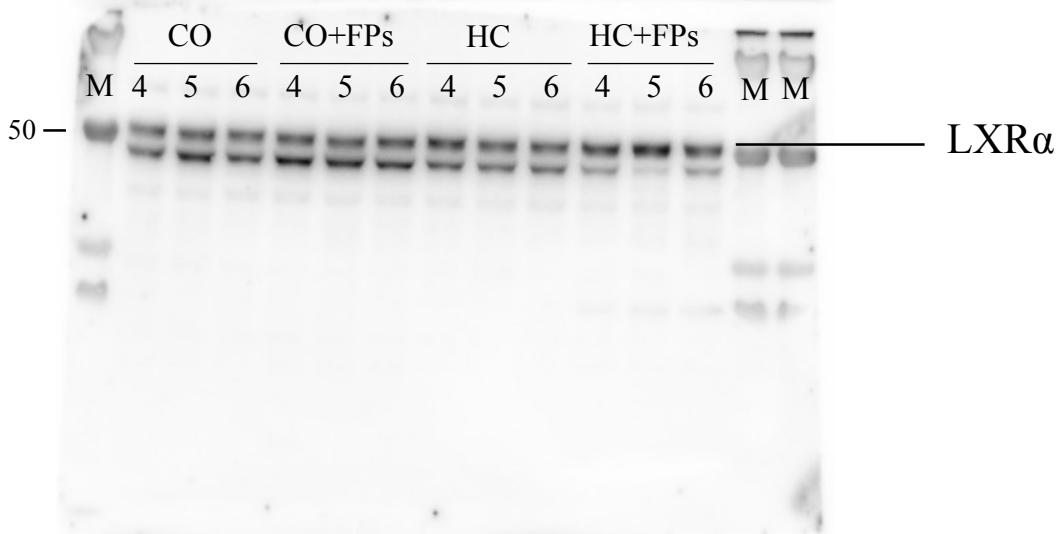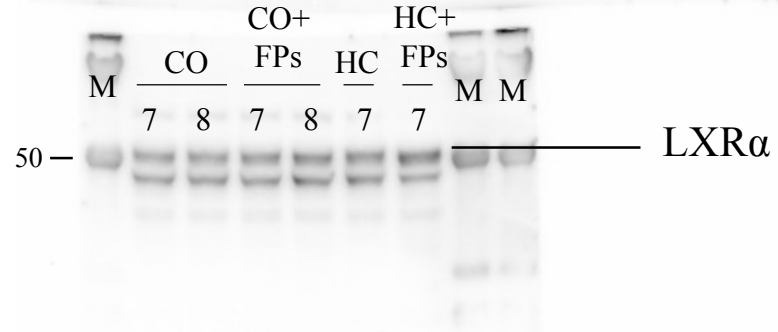

(kDa)

$\beta$ -actin

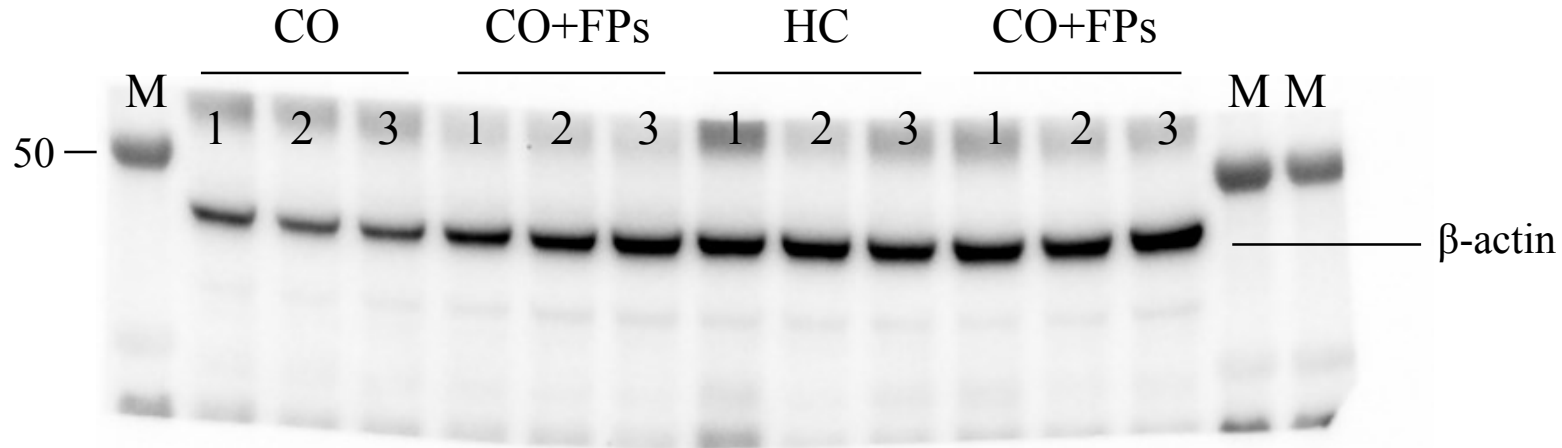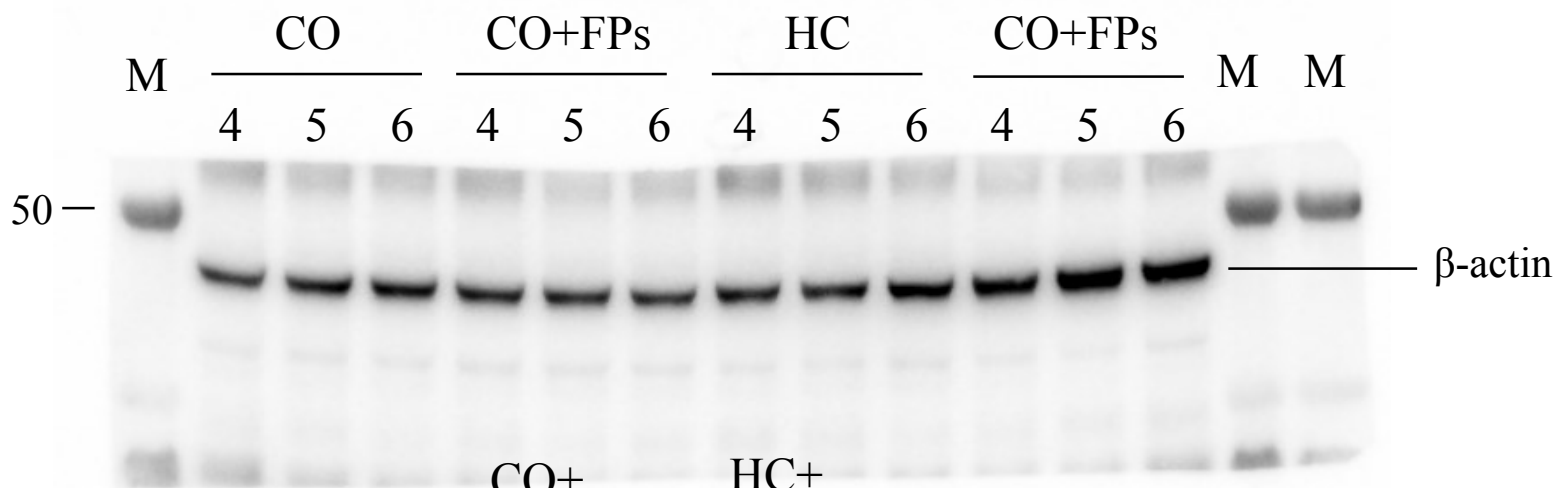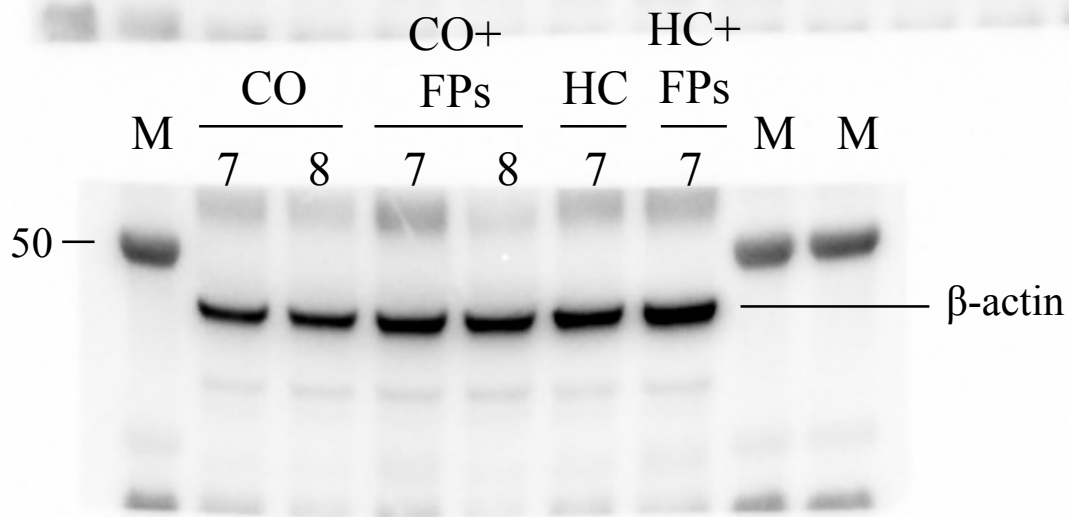

(kDa)

$\beta$ -actin

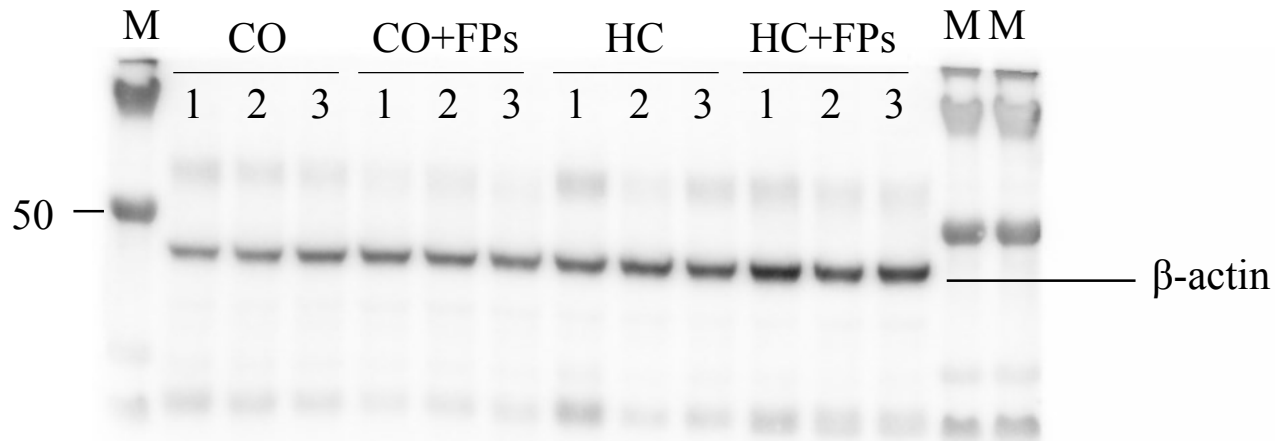

50—

$\beta$ -actin

(kDa)

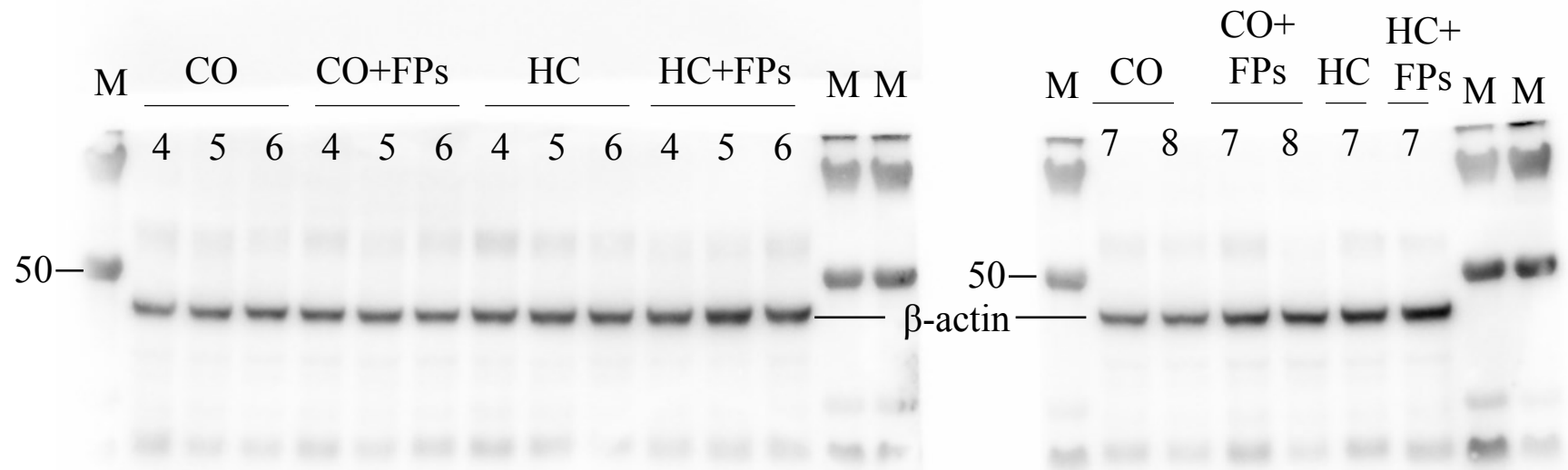

50—

$\beta$ -actin

Supplement: Supplemental Information 1 [file peerj-07-7671-s001.zip › Figure1_raw_data_band.pdf]
